# Relation between biomarkers and suicide attempts in patients with schizophrenia

**DOI:** 10.1192/j.eurpsy.2024.293

**Published:** 2024-08-27

**Authors:** A. Garcia Fernandez, C. Martínez-Cao, M. Couce-Sánchez, L. González-Blanco, P. Sáiz, P. García-Portilla

**Affiliations:** ^1^University of Oviedo, Oviedo, Spain

## Abstract

**Introduction:**

An increased risk of suicide has been reported by psychiatric patients, including schizophrenia^1^. Numerous evidence suggests alterations in the grade of pro-inflammatory impact on suicidal behavior^2^, and this relation has been shown in patients with mood or anxious disorders^3,4^. However, the grade of inflammation impact suicidal behavior in patients with schizophrenia has hardly been investigated.

**Objectives:**

Identify peripheral blood biomarkers of suicidal behavior in patients with schizophrenia, including inflammatory and lipid profile parameters.

**Methods:**

Secondary analysis of a cross-sectional study. Sample: 254 patients with schizophrenia, aged 18-72. Assessments: ad-hoc demographic and clinical questionnaire, PANSS, CDS, CAINS, PSP. Inflammatory and lipid parameters: C-reactive protein (PCR), interleukin 6 (IL-6); high-density lipoprotein cholesterol (HDL-C), low density lipoprotein cholesterol (LDL-C), total cholesterol (TC), triglyceridaemia (TG). Statistical analysis: Correlations, T Student, U Mann-Withney and lineal regression.

**Results:**

Mean age: 40.49 (13.10). Men: 64.2%.

No statistically significant differences were found between patients with suicide attempts and those without in any of the inflammatory or lipid parameters (p>0.05). However, differences were found in terms of suicide attempts (yes/no) in the PANSS negative (T=-2.217; p=0.028) and PANSS general psychopathy (T=-4.224; p< 0.001), in depressive symptoms (T =-6.967; p< 0.001), and the MAP subscale of the CAINS (T= -3.741; p<0.001).

Among patients with suicide attempts (n=42; 16.52% of the sample) (mean=1.90; sd=1.73; Range:1-7), statistically significant correlations were found with PCR (r=0.309; p=0.046), but not with cytokines and lipid parameters. On the other hand, no correlations were found with age, sex, length of illness, and any of the clinical scales.

A multiple linear regression was performed considering the number of suicide attempts as the dependent variable and as independent variables, age, sex, and those that were significant in the bivariate analysis (PCR).

A predictive model was found that explains 9.60% of the variance of number of suicide attempts (F = 4.224; p < 0.001). The variable that entered the model was PCR (β= 0.309; p=0.046).

**Conclusions:**

The increase in inflammation (manifested by the elevation of PCR) is related to an increase in the number of suicides. On the contrary, no correlations were found with lipid parameters or interleukins.

**Disclosure of Interest:**

None Declared

